# Supplementary material for: Dopaminergic neurons write and update memories with cell-type-specific rules
Source: eLife. 2016 Jul 21;5:e16135. doi: 10.7554/eLife.16135 (PMC4987137; doi:10.7554/eLife.16135)
Supplement: Supplementary file 1. — The first page shows a side view of the apparatus with a parts list. On the second page, a 3D model of the apparatus is shown which can be rotated and the visualization of each part can be individually turned on or off. DOI: http://dx.doi.org/10.7554/eLife.16135.016 [file elife-16135-supp1.pdf]

REVISION HISTORY				
ZONE	REV	DESCRIPTION	DATE	APPROVED
	-	INITIAL RELEASE	8/21/2015	HHMI\\negrashovi
	A	TRANSDUCERS WERE REMOVED	12/10/2015	HHMI\\negrashovi
	B	ACRYLIC INSERT	2/26/2016	HHMI\\negrashovi

NOTES:
- FASTENING IS NOT SHOWN
- PARTS J005342 AND J001841 ARE 3D PRINTED IN HOUSE
- PART J002451 IS GLUED TO J002447

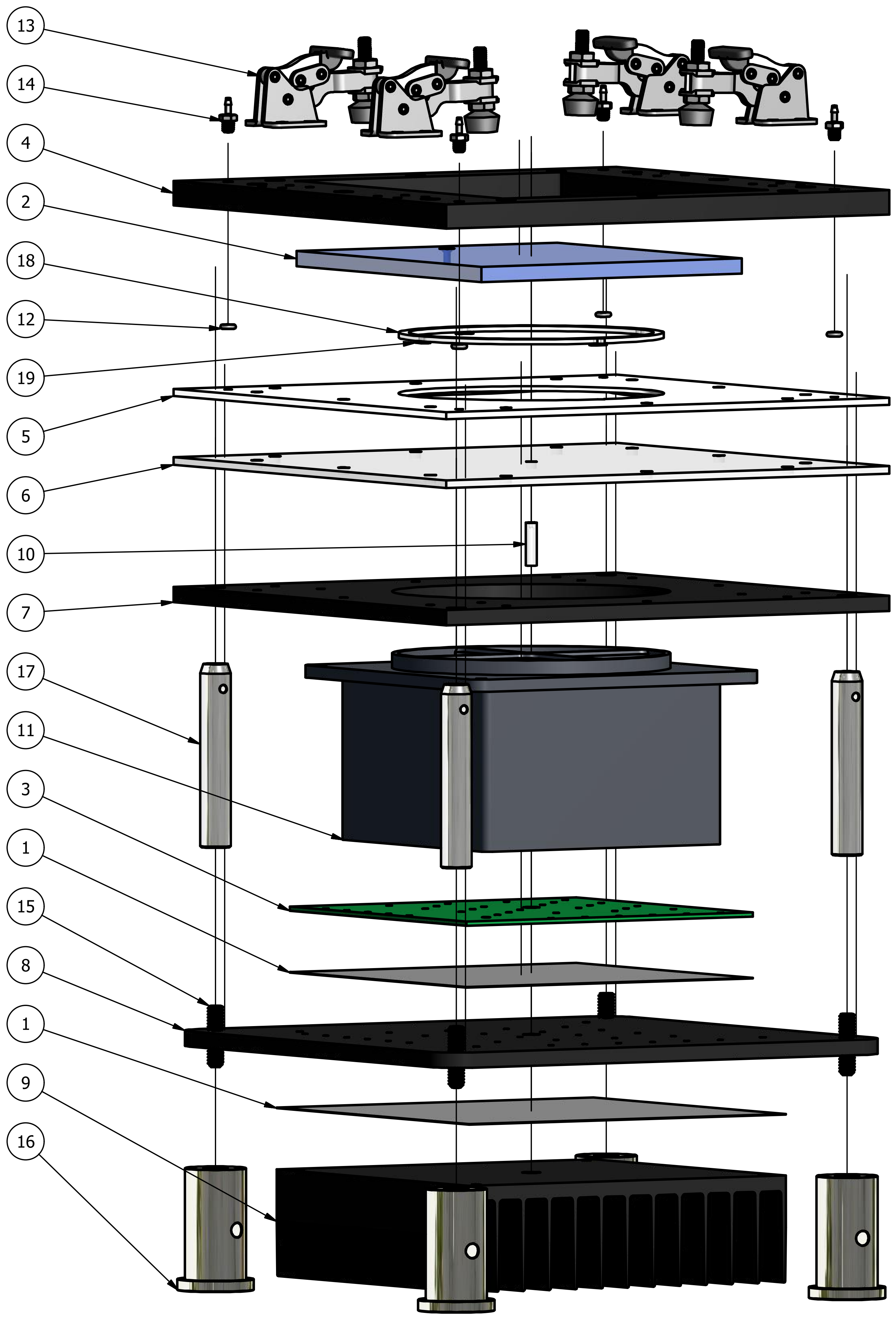

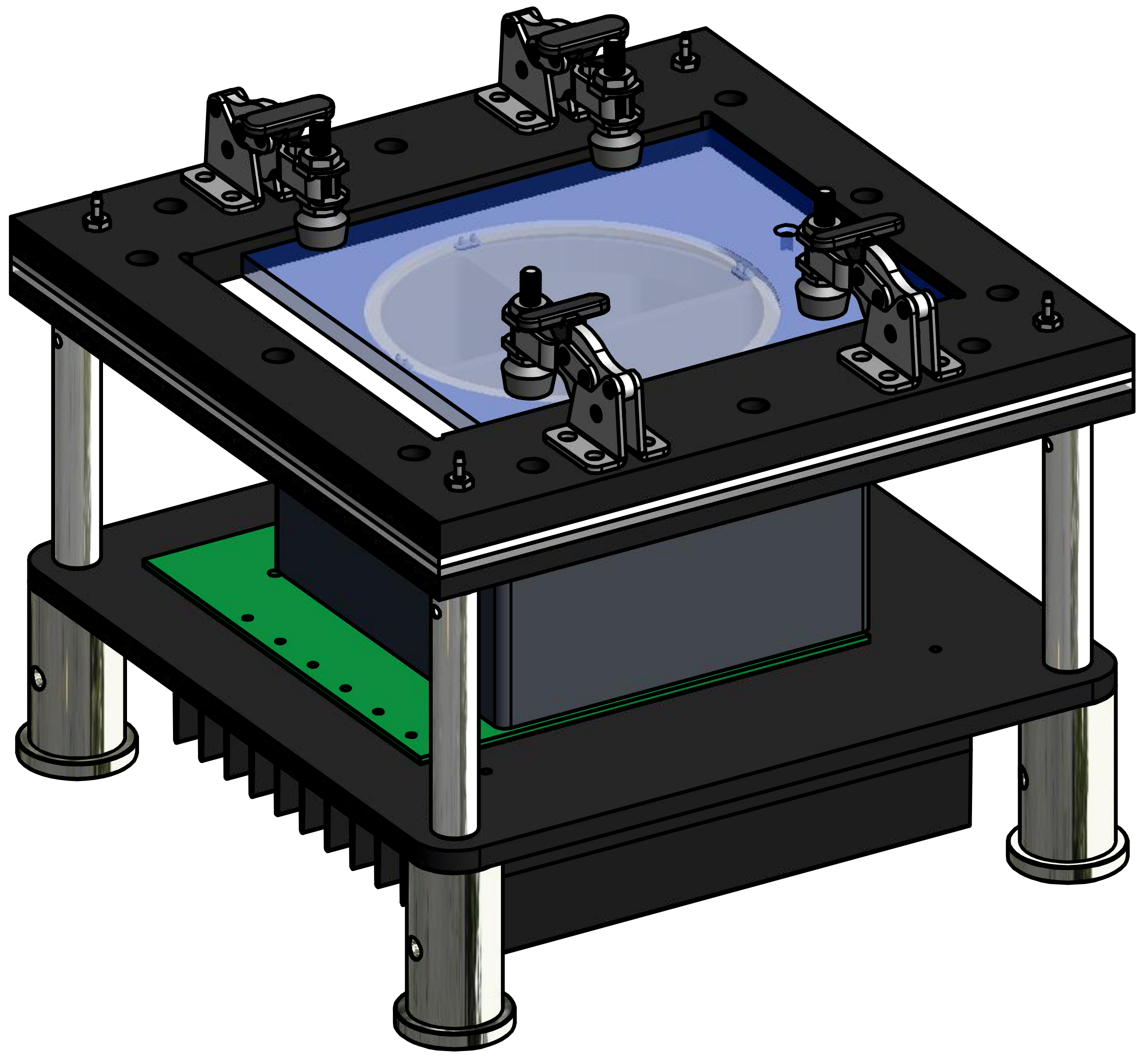

ITEM	QTY	FILE NAME	REV	PART NUMBER	VENDOR	DESCRIPTION	A.I. PART #	A.I. VENDOR
1	2	*Varies*	-	COMMON	DIGIKEY	THERMAL PAD	N/A	N/A
2	1	CHAMBER COVER GLASS.ipt	-	J001843	HHMI: JFRC - ID&F	CHAMBER COVER GLASS	N/A	N/A
3	1	NEW PCB.ipt	-	J002287	HHMI: JFRC - ID&F	TWO COLORS BACKLIGHT	N/A	N/A
4	1	CLAMPS MOUNT.ipt	-	J002445	HHMI: JFRC - ID&F	CHAMBER SPACER	N/A	N/A
5	1	CHAMBER SPACER.ipt	A	J002446	HHMI: JFRC - ID&F	CHAMBER SPACER	N/A	N/A
6	1	CHAMBER BOTTOM.ipt	-	J002447	HHMI: JFRC - ID&F	CHAMBER BOTTOM GLASS	N/A	N/A
7	1	ARENA SUPPORT.ipt	B	J002448	HHMI: JFRC - ID&F	ARENA SUPPORT	N/A	N/A
8	1	PCB SUPPORT.ipt	C	J002449	HHMI: JFRC - ID&F	PCB SUPPORT	N/A	N/A
9	1	AAVID THERMALLOY_60140_HEAT SINK EXTRUSION.ipt	-	J002450	HHMI: JFRC - ID&F	HEAT SINK EXTRUSION ALTERED	60140	AAVID THERMALLOY
10	1	VACUUM FITTING.ipt	-	J002451	HHMI: JFRC - ID&F	VACUUM FITTING	N/A	N/A
11	1	LIGHT SEPARATOR.ipt	-	J005342	HHMI: JFRC - ID&F	LIGHT SEPARATOR	N/A	N/A
12	4	MCMASTER_9452K14_O-RING DASH N 006.ipt	-	1163N104	MCMASTER	VITON CLEAN ROOM O-RING DASH # 006	N/A	N/A
13	4	MCMASTER_5004A820_TOGGLE CLAMP.ipt	-	5004A11	MCMASTER	TOGGLE CLAMP	N/A	N/A
14	4	BARBED ADAPTER.ipt	-	FITM328	NRESEARCH	BARBED ADAPTER 1/16 TUBE ID	N/A	N/A
15	4	MCMASTER_92313A542_CUP POINT SET SCREW 1%4-20 LENGTH 1 IN.ipt	-	92313A542	MCMASTER	CUP POINT SET SCREW 1/4-20 LENGTH 1 IN	N/A	N/A
16	4	THORLABS_RS2P_Ø1 IN x 2 IN OPTICAL POST.ipt	-	RS2P	THORLABS	Ø1 IN x 2 IN OPTICAL POST	N/A	N/A
17	4	THORLABS_TR3_Ø0.5 IN x 3 IN OPTICAL POST.ipt	-	TR3	THORLABS	Ø0.5 IN X 3 IN OPTICAL POST	N/A	N/A
18	1	RING.ipt	-	J005536	HHMI: JFRC - ID&F	RING FOR ROUND INSERT	N/A	N/A
19	4	SUPPORT.ipt	-	J005537	HHMI: JFRC - ID&F	SUPPORT FOR ROUND INSERT	N/A	N/A

PARTS LIST

NOTICE:
INFORMATION CONTAINED IN THIS DOCUMENT OR ANY REPRODUCTION THEREOF, IS PROPRIETARY INFORMATION AND PROPERTY OF HOWARD HUGHES MEDICAL INSTITUTE. IT SHALL NOT BE DISCLOSED, COPIED, DUPLICATED OR USED FOR MANUFACTURE, PRODUCTION OR PROCUREMENT, WITHOUT THE EXPRESS WRITTEN PERMISSION OF HOWARD HUGHES MEDICAL INSTITUTE.

(UNLESS SPECIFIED OTHERWISE)
PRIMARY UNITS: INCHES
[SECONDARY UNITS]: MILLIMETERS

PRIMARY TOLERANCES:	
X.X	± 0.020
X.XX	± 0.010
X.XXX	± 0.005
X.XXXX	± 0.0005
ANGULAR	± 0.5 DEG

- DO NOT SCALE DRAWING -
THIRD ANGLE PROJECTION:

hhmi Howard Hughes Medical Institute

hhmi janelia Research Campus

OLFACTORY ASSAY ARENA.iam

SIZE	PART NUMBER	REV	SHEET
C	J005341	B	1 OF 2

4 3 2 1

3D VIEW. CLICK AND DRAG WITH LEFT BUTTON OF MOUSE

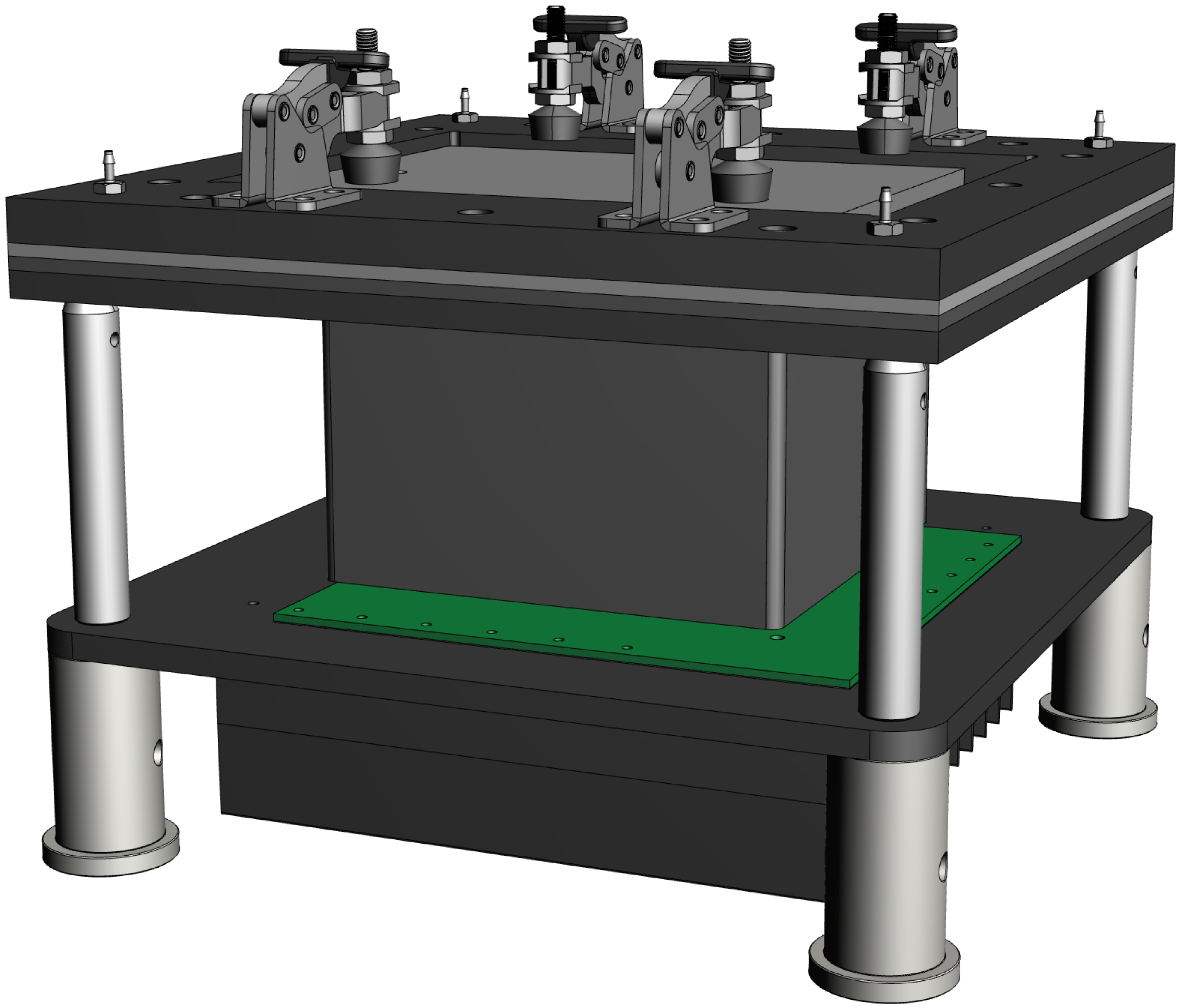

NOTICE:
INFORMATION CONTAINED IN THIS DOCUMENT OR ANY REPRODUCTION THEREOF, IS PROPRIETARY INFORMATION AND PROPERTY OF HOWARD HUGHES MEDICAL INSTITUTE. IT SHALL NOT BE DISCLOSED, COPIED, DUPLICATED OR USED FOR MANUFACTURE, PRODUCTION OR PROCUREMENT, WITHOUT THE EXPRESS WRITTEN PERMISSION OF HOWARD HUGHES MEDICAL INSTITUTE.

(UNLESS SPECIFIED OTHERWISE)
PRIMARY UNITS: INCHES
[SECONDARY UNITS]: MILLIMETERS

PRIMARY TOLERANCES:

X.X	± 0.020
X.XX	± 0.010
X.XXX	± 0.005
X.XXXX	± 0.0005
ANGULAR	± 0.5 DEG

- DO NOT SCALE DRAWING -
THIRD ANGLE PROJECTION:

hhmi Howard Hughes Medical Institute
hhmi janelia Research Campus

SIZE	PART NUMBER	REV	SHEET
C			2 OF 2

4 3 2 1
